# Graphene/Carbon Nanotube Hybrid Nanocomposites: Effect of Compression Molding and Fused Filament Fabrication on Properties

**DOI:** 10.3390/polym12010101

**Published:** 2020-01-04

**Authors:** Sithiprumnea Dul, Luiz Gustavo Ecco, Alessandro Pegoretti, Luca Fambri

**Affiliations:** 1 Department of Industrial Engineering and INSTM Research Unit, University of Trento, 38123 Trento, Italy; sithiprumnea.dul@unitn.it (S.D.); alessandro.pegoretti@unitn.it (A.P.); 2 Department of Mechanical Engineering, Federal University of Santa Catarina—UFSC, Florianópolis 88040-900, SC, Brazil; luiz.ecco@labmat.ufsc.br

**Keywords:** conductive composites, carbon nanotubes, graphene, electromagnetic interference shielding, mechanical properties

## Abstract

The present work reports on the production and characterization of acrylonitrile butadiene styrene (ABS) hybrid nanocomposite filaments incorporating graphene nanoplatelets (GNPs) and carbon nanotubes (CNTs) suitable for fused filament fabrication (FFF). At first, nanocomposites with a total nanofiller content of GNP and/or CNT of 6 wt.% and a GNP/CNT relative percentage ratio of 0, 10, 30, 50, 70, and 100 were produced by melt compounding and compression molding. Their mechanical, electrical resistivity, and electromagnetic interference shielding effectiveness (EMI SE) properties were evaluated. The hybrid nanocomposites showed a linear increase in modulus and decrease in strength as a function of GNP content; on the other hand, the addition of CNT in hybrid nanocomposites determined a positive increase in electrical conductivity, but a potentially critical decrease of melt flow index. Due to the favorable compromise between processability and enhancement of performance (i.e., mechanical and electrical properties), the hybrid composition of 50:50 GNP/CNT was selected as the most suitable for the filament production of 6 wt.% carbonaceous nanocomposites. EMI SE of ABS-filled single CNT and hybrid GNP/CNT nanofillers obtained from compression molding reached the requirement for applications (higher than −20 dB), while slightly lower EMI SE values (in the range −12/−16 dB) were obtained for FFF parts dependent on the building conditions.

## 1. Introduction

Polymer nanocomposites with carbonaceous nanomaterial reinforcements are extensively investigated due to their remarkable performance including mechanical, electrical, and thermal properties. In particular, the use of nanomaterials can offer extraordinary structural and functional properties of polymers that can be tailored for broad application in many fields, but sometimes requires an adequate optimization of material processability. The nanocomposites could help the development of lightweight structural materials with functionalities that can be utilized for electronic components, micro-batteries, circuits, and electromagnetic shielding [1,2,3,4,5].

The extensive global development of novel electronic devices in many fields such as industrial, household, medical, science, military, and telecommunication cause electromagnetic pollution. Therefore, electromagnetic interference (EMI) is a major concern in modern society because it could affect the normal functionality of electronic devices and human health. Consequently, shielding is necessary for preventing electronics from undesired electromagnetic radiations associated with strategic systems utilized in the aerospace industry, aircraft, automobiles, flexible electronics, and wearable devices [6,7,8,9]. Conductive materials that are employed in shielding electromagnetic waves are typically metals and many forms of carbonaceous materials, e.g., carbon black, carbon nanotube (CNT), and graphite/graphene (GNP). However, utilizing metals in EMI shielding applications could cause drawbacks including an increase in weight, electrochemical corrosion, and more expensive processing methods [10,11,12,13]. Conductive polymers gained popularity in EMI shielding applications due to their relatively low cost, light weight, and easy processing. Because neat polymers possess poor EMI shielding properties, they are often compounded with conductive nanofillers via conventional processing methods (e.g., solvent casting [14,15,16], melt mixing followed by compression molding [17,18,19,20], and extrusion and injection [21]), as well as via additive manufacturing [2,22,23].

In the last decade, additive manufacturing (AM), also known as three-dimensional (3D) printing, was largely developed in both industry and research centers. An AM machine reads in data from a digital model and lays down or adds successive layers of liquid, powder, or sheet material, in a layer-upon-layer fashion, to fabricate a 3D object. This technology has several advantages such as the possibility to fabricate a final part without using auxiliary tool/molds and building complex geometric parts which are difficult via conventional methods [24,25,26,27]. Generally, additive manufacturing is more preferable for the relatively small-scale productions and it exhibits evident advantages with respect to subtractive manufacturing methodologies because of no residue after the process. There are several popular methods for AM such as stereolithography (SLA), using a focused ultraviolet (UV) laser beam to photopolymerize the uncured resin layer by layer, selective laser sintering (SLS), using a scanning laser beam to sinter the powdered materials at the cross-sections, and fused filament fabrication (FFF), using a small extruder to melt and deposit a thermoplastic filament. The FFF technique, which is most dominant in AM methods due to its low cost and easy use, requires fewer post-processing steps, and a large variety of materials can be used. The process is a simple extrusion of filaments in the molten state through a heated nozzle (350–600 μm) to create 3D objects via layer-by-layer deposition in the horizontal plane (*XY* plane) [26].

The development of nanocomposite materials could be a way to improve the properties of components produced by FFF. Some studies reported that combining two nanofillers (e.g., carbon nanotubes and graphene) could induce a synergistic effect in various matrices such as epoxy [28,29,30], polylactic acid (PLA) [31,32,33], and thermoplastic polyurethanes (TPUs) [34]. The mechanical properties and the thermal and electrical conductivities of double-filler nanocomposites could exhibit higher values than those of single-filler nanocomposites, due to the formation of a co-supporting network of both fillers. However, the synergistic effect of nanofillers is not completely understood scientifically. Very recent works reported hybrid graphene nanoplatelet (GNP)/carbon nanotube (CNT) nanofillers for the FFF technique in different matrices, such as PLA [31,32,33] and polyether ether ketone (PEEK) [35]. PLA-carbon based nanocomposites derived from the FFF technique were also investigated in terms of electrical and thermal conductivity and electromagnetic shielding behavior. In particular, taking into consideration the T_g_ of the PLA matrix, a specific approach to compensate for the temperature effect on the resistivity of a PLA conductive sample in the range 20–50 °C was discussed [36]. In order to enlarge the number of application fields with high-performance properties, especially for high temperature, a different approach was recently proposed with processing and 3D printing of PEEK filled with GNP/CNT up to 380 °C. Tensile properties, electrical and thermal conductivity, and tribological properties of 3D-printed nanocomposites were properly studied and considered for aerospace applications [35]. However, no research reported a hybrid GNP/CNT nanofiller in an acrylonitrile butadiene styrene (ABS) matrix, whose temperature range of applications goes up to 80 °C. In particular, the electromagnetic shielding behavior of 3D-printed samples remains to be investigated.

Our previous research focused on the development of nanocomposite filaments within carbonaceous nanofillers (i.e., graphene, carbon nanotube, and carbon black) suitable for the FFF technique with characterizations of the structure and physical properties [22,23,37,38,39]. Both graphene and carbon nanotubes were successfully utilized in the FFF process, determining positive effects at different levels [37,39]. The incorporation of CNT provided not only a high increase in electrical conductivity, but also a severe increase in the viscosity of materials that consequently required a high-temperature FFF process (280 °C). On the other hand, GNP was found not effective for resistivity reduction, but GNP/ABS nanocomposites could maintain better processability due to their relatively higher melt flow index. In our previous work, we concluded that proper combinations of CNT and GNP at a suitable fraction (e.g., 6 wt.% in the hybrid) could offer a possible compromise between relatively easy processability and an acceptable enhancement of performance (i.e., mechanical and specific electrical properties) for applications where polymeric materials with low electrical resistivity are required [40].

The present work focuses on the preparation and characterization of ABS nanocomposites based on a hybrid composition of GNP and CNT, suitable for FFF 3D printing. In a preliminary investigation, the total amount of nanofiller of nanocomposites fixed at 6 wt.% with the variation of GNP/CNT relative ratio was produced by compression molding. Optimal compositions were selected for extrusion and the FFF technique, and the produced single-filler and hybrid nanocomposites were characterized under mechanical, electrical, and electromagnetic interference shielding testing.

## 2. Materials and Methods 

### 2.1. Materials

Acrylonitrile butadiene styrene (ABS) Sinkral^®^ F322 (melt flow rate of 14 cm^3^/10 min at 220 °C/10 kg; density of 1.04 g/cm^3^) used in this study was supplied by Versalis S.p.A. (Mantova, Italy) [41]. Before processing, ABS pellets were dried at 80 °C in a vacuum oven for at least 2 h. The selected carbon nanoparticles, graphene nanoplatelets and carbon nanotubes, are presented in Table 1 with details of their dimensions and physical characteristics according to the manufacturer datasheet. 

The basic quality assessment data of nanofillers are available in the technical data sheets for GNP [42] and CNT [43], as well as in some literature publications. In particular, Raman spectra and X-ray Diffraction (XRD) characterization were reported for GNP [42,45] and CNT [46]. TEM images of both GNP and CNT were also illustrated in our previous publication [40]. Moreover, TEM micrographs of MWCNT-NC7000 in an ABS nanocomposite evidenced a certain level of orientation in filaments [47].

### 2.2. Material Processing and Sample Preparation

#### 2.2.1. Compounding

Various GNP/CNT ratio nanofillers at a total concentration fixed at 6 wt.%, as detailed in Table 2, were mixed with neat ABS by melt compounding in a counter-rotating Thermo-Haake Polylab Rheomix internal mixer (Thermo Haake, Karlsruhe, Germany) at a temperature of 190 °C, with a rotor speed of 90 rpm, for 15 min. Neat ABS was also processed under the same conditions.

#### 2.2.2. Compression Molding (CM)

Compounded batches of about 50 g were produced for each composition. The compounded materials were hot-pressed at 190 °C in a Carver Laboratory press (Carver, Inc., Wabash, IN, USA) for 10 min under a pressure of 3.9 MPa to obtain square plates with dimensions 160 × 160 × 1.2 mm^3^ (for mechanical and resistivity tests) and 120 × 120 × 2 mm^3^ (for electromagnetic interference shielding effectiveness (EMI SE) analysis).

#### 2.2.3. Filament Extrusion

**Figure 1:**
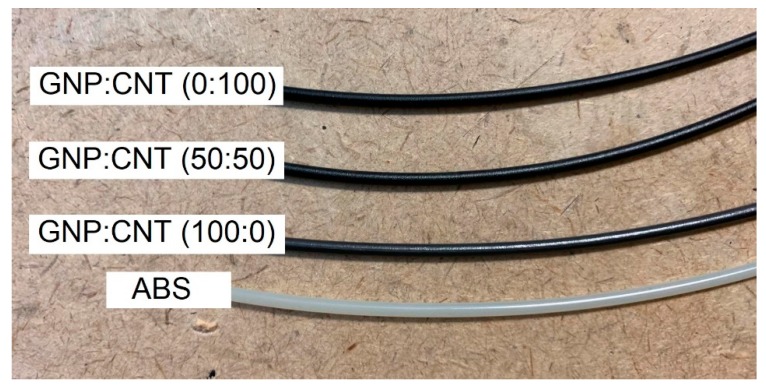
Filaments of neat acrylonitrile butadiene styrene (ABS), and graphene nanoplatelet (GNP)/multi-walled carbon nanotube (CNT) (100:0), GNP/CNT (50:50), and GNP/CNT (0:100) nanocomposites at 6 wt.% of nanofiller.

Compounded materials were also used to feed a Thermo Haake PTW16 intermeshing co-rotating twin-screw extruder produced by Thermo Haake, Karlsruhe, Germany (screw diameter = 16 mm; *L*/*D* ratio = 25; rod die diameter 1.80 mm). The processing temperature profile gradually increased from *T*_1_ = 180 °C to *T*_2_ = 205 °C, *T*_3_ = 210 °C, *T*_4_ = 215 °C, and *T*_5_ = 220 °C (rod die). The screw rotation speed was fixed at 5 rpm, and collection rate was regulated (about 1.0 m/min) by using a take-up unit Thermo Electron Type 002-5341 in order to obtain a final diameter of the extruded filament of 1.75 ± 0.05 mm (see Figure 1).

#### 2.2.4. FFF Printed Sample Preparation

The 3D-printed specimens were manufactured by a prototype 3D printer for high-temperature processing, Sharebot HT Next Generation desktop 3D printer (Sharebot NG, Nibionno, LC, Italy), fed with the filaments extruded as described in the previous paragraph. The 3D-printed samples were built in three configurations, horizontal concentric (HC), horizontal 45° angle (H45), and perpendicular concentric (PC), as detailed in our previous publication [39]. In summary, all the specimens were 3D-printed using the following printing parameters: object infill 100%; no raft; nozzle diameter 0.40 mm; bed temperature 110 °C; layer height 0.20 mm; raster angle (0°/0°) for HC and PC and raster angle (+45°/−45°) for H45; infill speed 40 mm/s for HC and H45 and 16 mm/s for PC specimens. The 3D-printed parts were manufactured at a nozzle temperature of 280 °C for the CNT nanocomposite, and 250 °C for the ABS matrix, GNP, and hybrid GNP/CNT nanocomposites.

### 2.3. Testing Techniques

#### 2.3.1. Scanning Electron Microscopy (SEM)

Nanocomposites were fractured in liquid nitrogen and their representative fracture surfaces at different levels of magnification were observed by a Carl Zeiss AG Supra 40 field-emission scanning electron microscope (FE-SEM) (Carl Zeiss AG, Oberkochen, Germany) at an acceleration voltage of 5 kV.

#### 2.3.2. Melt Flow Index (MFI)

The MFI measurements were performed according to the ASTM D 1238 standard (procedure A), using a Kayeness Co. model 4003DE capillary rheometer (Morgantown, PA, USA), at 220 °C/10 kg with a pre-heating and compaction time of about 5 min on samples with a mass of about 5 g cut from compression molded plates.

#### 2.3.3. Tensile Test

Uniaxial tensile tests were performed at room temperature using an Instron^®^ 5969 electromechanical tester (Norwood, MA, USA) equipped with a 50-kN load cell. Strength and strain at break values were determined at a crosshead speed of 10 mm/min as an average of at least three replicates. Three categories of samples were investigated: (1) compression molded (CM) samples ISO 527 type 1BA dumbbell (gauge length 30 mm; thickness 1.2 mm); (2) filaments (gauge length 100 mm; diameter 1.75 mm); (3) 3D-printed samples (HC, H45, and PC), ISO 527 type 5A dumbbell (gauge length 25 mm; thickness 2 mm).

The elastic modulus of CM and 3D-printed specimens was evaluated at a crosshead speed of 1 mm/min using an electrical extensometer Instron^®^ model 2620-601 (Norwood, MA, USA) with a gauge length of 12.5 mm. The elastic modulus of the filament was measured at a crosshead speed of 10 mm/min without an extensometer with a gauge length of 100 mm, taking the system compliance into account. According to the ISO 527 standard, the elastic modulus was determined as a secant value between strain levels of 0.05% and 0.25%.

#### 2.3.4. Electrical Resistivity Test

For samples with an electrical resistivity higher than 10^7^ Ω∙cm, the volume resistivity ρ was determined according to ASTM D257 using a Keithley 6517A electrometer/high-resistance meter (Beaverton, OR, USA) and an 8009 resistivity test fixture at room temperature. In this test, a constant voltage of 100 V was applied to square samples of 64 × 64 × 1.2 mm^3^.

For moderately conductive materials (ρ < 10^7^ Ω∙cm), the electrical resistivity test was measured according to the ASTM D4496-04 standard with a four-point contact configuration. Each specimen was subjected to a voltage of 5 V generated by a direct current (DC) power supply IPS303DD produced by ISO-TECH (Milan, Italy). Simultaneously, the current flow on the samples was recorded between external electrodes by using an ISO-TECH IDM 67 Pocket Multimeter electrometer (ISO-TECH, Milan, Italy). Compression molding (CM), filaments, and 3D-printed samples were tested with a length of 25 mm and different cross-section (rectangular specimens 6 × 1.2 mm^2^ for CM and 6 × 2 mm^2^ for 3D-printed sample; diameter of 1.75 mm for filament). Average values of resistivity from at least three replicates were reported. A conductive silver paint was applied to the surface of 3D-printed samples in order to obtain good electrical contact. The electrical volume resistivity of the samples was determined by Equation (1).

(1) $$\rho =R\times \frac{A}{L}$$

where *R* is the electrical resistance, *A* is the is the cross-section of the specimen, and *L* is the distance between the internal electrodes (i.e. 3.69 mm). All the reported electrical conductivity and resistivity values were volume electrical conductivity and volume resistivity, taking into account the thickness of samples.

#### 2.3.5. Electromagnetic Interference Shielding Effectiveness (EMI SE)

The electromagnetic interference shielding capabilities of CM and 3D-printed samples were measured using an Agilent Technology PNA series network analyzer (N5230C Agilent PNA-L, Santa Clara, CA) and a standard rectangular waveguide in the X-band frequency range (8.2–12.4 GHz). The analysis was performed on compression molded and FFF samples with a dimension 45 × 45 × 2 mm^3^, and the S-parameters (S_11_, S_22_, S_12_, S_21_) were recorded over the X-band frequency range, as detailed in the literature [23,44]. The contributions of reflection (SE_R_) and absorption (SE_A_) to the total EMI SE of the composites were investigated, while the effect of multiple reflections (SE_M_) was neglected. At least three specimens were tested for each sample, and the standard deviations were calculated.

## 3. Results and Discussions

### 3.1. Melt Flow Index and Morphology

**Figure 2:**
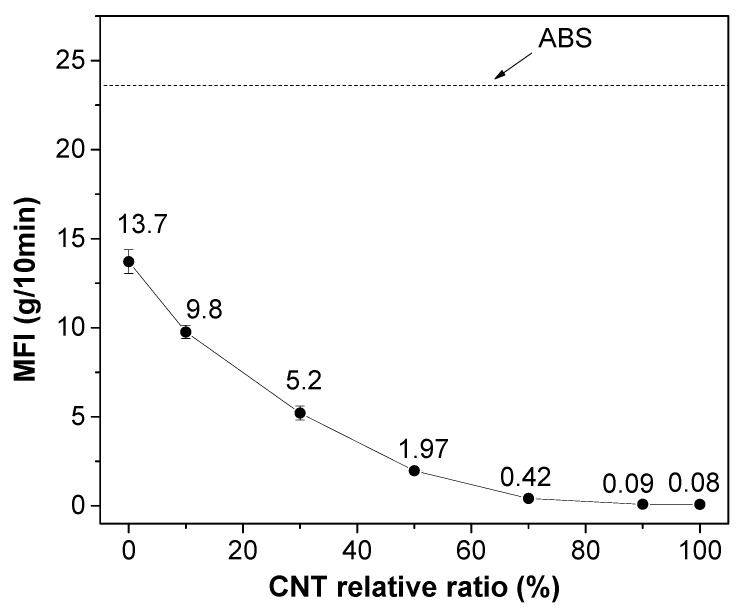
Melt flow index (MFI) (220 °C/10 kg) of 6 wt.% GNP/CNT nanocomposites with different CNT contents.

The flow properties of hybrid nanocomposite formulations at a fixed total amount of filler (6 wt.%) as a function of the different fraction ratios is presented in Figure 2. The MFI values of nanocomposites significantly decreased with the CNT content. By optimizing the enhancement of properties (mechanical and electrical properties) and processability, the selected composition of 50:50 of 6 wt.% of hybrid nanocomposite was extruded into filaments for FFF. It is worthwhile to note that the GNP/CNT (50:50) nanocomposite could be 3D-printed at 250 °C, whereas the GNP/CNT (0:100) nanocomposite required a processing temperature of 280 °C.

**Figure 3:**
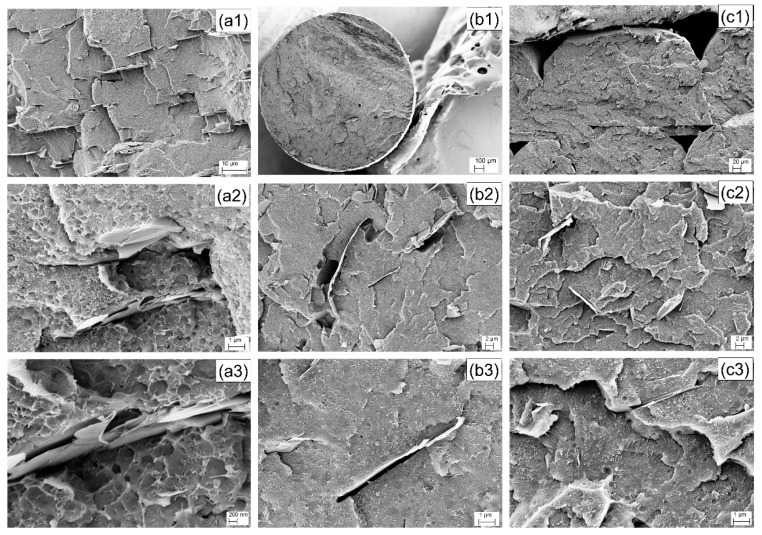
SEM micrographs at increasing magnification of GNP/CNT (50:50) nanocomposites produced by compression molding (**a1–a3**), filaments (**b1**–**b3**), and fused filament fabrication (FFF) horizontal concentric (HC) samples (**c1–c3**).

The SEM pictures of the fracture surface of GNP/CNT (50:50) nanocomposites as obtained from various processes including compression molding, filament extrusion, and 3D printing, are shown in Figure 3. SEM figures of compression molded plates (see Figure 3a1–a3) evidence a poor adhesion level between graphene and ABS. The effect of the two processing steps of compounding and extrusion, and the quality of carbon nanotubes and graphene dispersion into the ABS matrix were evaluated from the fracture surface of the GNP/CNT (50:50) nanocomposite filament by SEM analysis, as depicted in Figure 3b1–b3. Some small voids can be observed in Figure 3b1, and, at higher magnification, some microvoids can be evidenced near the filler, as documented by Figure 3b2. In particular, the graphene nanoplatelets appear to be oriented mostly perpendicular to the fracture plane of the filament, as depicted in Figure 3b2,b3. Similar to the filament, 3c1–c3 show that, in FFF, the graphene nanoplatelets for GNP/CNT (50:50) HC parts appear to be oriented mostly perpendicular to the fracture plane and, therefore, most likely oriented along the loading direction of dumbbell specimens. It can, therefore, be inferred that, during extrusion, the graphene nanoplatelets are forced to align along the extrusion direction of the filament, and, during the following FFF process, this orientation is then maintained in each single microfilament during the layer deposition.

### 3.2. Tensile Properties

**Figure 4:**
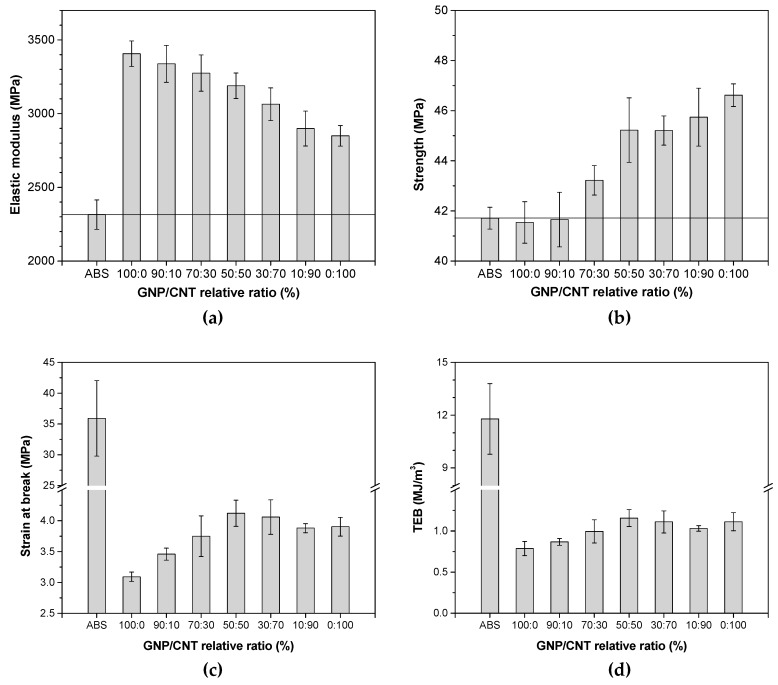
Tensile properties of ABS and various GNP/CNT hybrid nanocomposites with a total nanofiller amount of 6 wt.% produced by compression molding: (**a**) elastic modulus, (**b**) strength, (**c**) strain at break, and (**d**) tensile energy to break (TEB). Representative stress–strain curves are shown in Figure S1a (Supplementary Materials).

The tensile properties of hybrid nanocomposites with GNP/CNT at 6 wt.% obtained from compression molding are presented in Figure 4. The stiffness and strength of GNP/CNT hybrids at various mixture ratios were found superior to those of pure ABS. In particular, the elastic modulus of nanocomposites linearly increased with the amount of GNP content as shown in Figure 4a. For example, single-filler CNT and GNP nanocomposites evidenced elastic modulus improvements of 23% and 47%, respectively, while the hybrid nanocomposites with a ratio of 50:50 exhibited 37% improvement. On the other hand, the strength of nanocomposites increased with CNT content, as shown in Figure 4b. From Figure 4c,d, the strain at break and tensile energy to break of GNP/CNT (0:100) was slightly higher than that of GNP/CNT (100:0) nanocomposites, and the GNP/CNT (50:50) specimen revealed the highest strain at break in comparison with the other nanocomposites. Some studies reported a synergistic effect for hybrid carbon nanotube/graphene nanocomposites at a low concentration of 1 wt.% nanofiller [29,30,34]. On the contrary, our results suggest no evident synergistic effects on tensile modulus and strength, probably due to the higher concentration of nanofiller.

**Figure 5:**
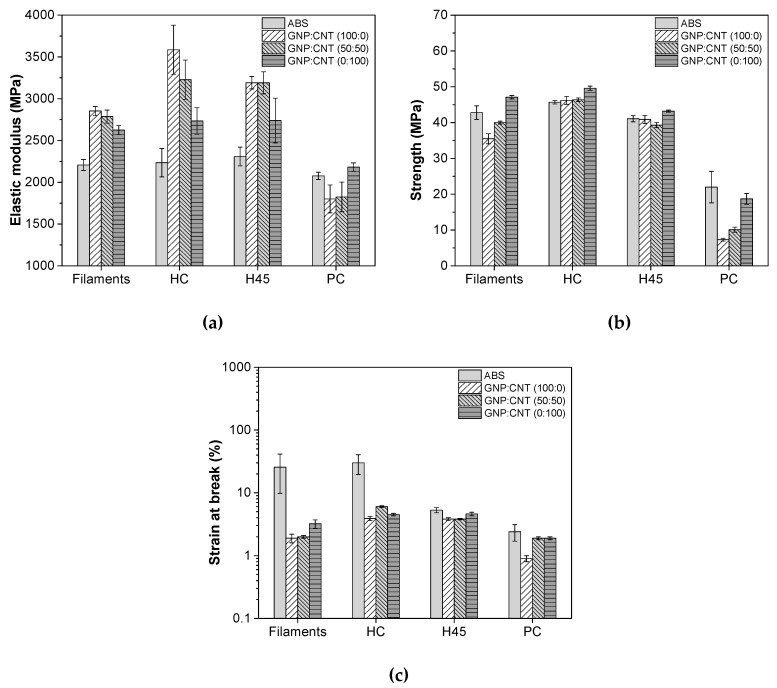
Quasi-static tensile properties of extruded filaments and FFF samples (HC, horizontal 45° angle H45, and perpendicular concentric (PC)) of ABS and its nanocomposite: (**a**) elastic modulus, (**b**) strength, and (**c**) strain at break. Representative stress–strain curves are shown in Figure S1b–e (Supplementary Materials).

The tensile properties of filament and 3D-printed samples including elastic modulus, strength, and strain at break are plotted in Figure 5. The ductility properties of nanocomposite filaments were significantly reduced with respect to neat ABS. In addition, it is worthwhile to note that the elastic modulus of the 50:50 hybrid nanocomposites was higher than that of single CNT nanocomposites, whereas the strength and strain at break of the hybrid composite filament were slightly lower than for pure CNT nanocomposites, and the sample was fractured before the yield point. The tensile properties of 3D-printed (HC and H45) samples showed a similar tendency to the sample produced by compression molding and to the filaments. In particular, Figure 5 evidences that the elastic modulus of hybrid nanocomposites of HC and H45 samples was further increased in comparison with the pure CNT nanocomposites; on the other hand, strength and strain at break of this material (50:50) were slightly reduced with respect to the single CNT nanocomposites. A different behavior was observed for FFF samples produced with a PC build orientation, for which the strength and strain at break of the GNP/CNT (50:50) hybrid and (0:100) were significantly reduced compared to neat ABS. Moreover, it should be noted that more brittle behavior of all the PC samples existed, almost independently of the GNP/CNT composition, due to the weakness of bond properties at the cross-sections. In previous work, PLA nanocomposites filled with various GNP/CNT ratios at 6 wt.% used in 3D printing and processing showed a synergistic effect on mechanical properties. In particular, hybrid nanocomposites evidenced a higher elastic modulus and hardness compared with single GNP and CNT nanocomposites at the same nanofiller concentration, hypothesizing that the long tortuous CNTs prevent GNP aggregation and bridge adjacent graphene, leading to a more efficient network and better reinforcing effects in the matrix [46].

### 3.3. Electrical Resistivity

**Figure 6:**
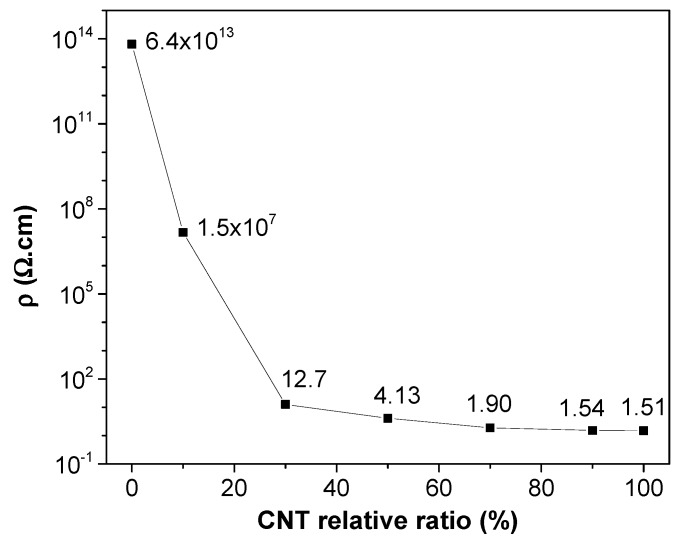
Electrical volume resistivity of 6 wt.% GNP/CNT nanocomposites at different CNT contents produced by compression molding.

Following the volume electrical measurements, the results of bulk resistivity measurements of the samples containing GNP and CNT nanofillers are reported in Figure 6. From this set of data, it is possible to understand that, upon increasing the CNT relative amount, the resistivity decreased with a nonlinear trend, because of the synergistic effect on electrical resistivity due to the presence of both nanofillers. With a total nanofiller content of 6 wt.%, the nanocomposite required at least a GNP/CNT ratio of 70:30 to have a low electrical resistivity of about 13 Ω∙cm (with an effective CNT content of 1.8 wt.%). On the other hand, single-filler nanocomposite samples loaded only with 2 wt.% of CNT exhibited an electrical resistivity of 30 Ω∙cm [40]; thus, the synergistic effect of GNP in hybrids appears evident. On the other hand, only a small synergistic effect in PLA nanocomposite was reported by Ivanov et al. [31], when combining GNP and CNT at ratios of 3 wt.% GNP/3 wt.% CNT and 1.5 wt.% GNP /4.5 wt.% CNT on electrical conductivity, with no effect on the thermal conductivity.

**Figure 7:**
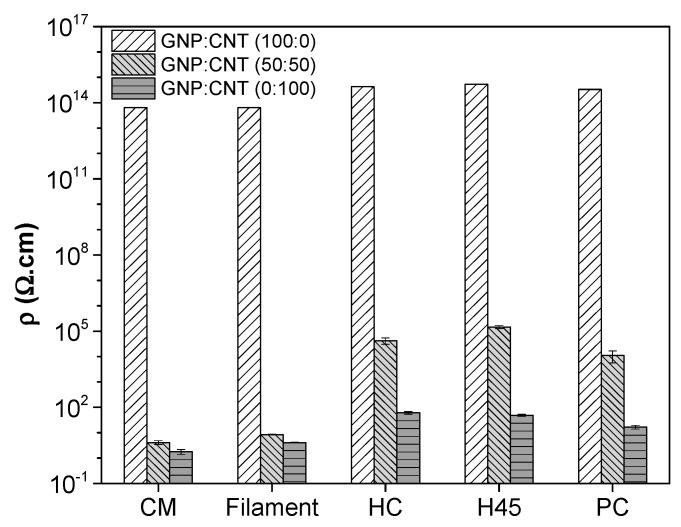
Electrical volume resistivity of GNP, CNT, and GNP/CNT nanocomposites with different processing: compression molding (CM), the filament, and FFF samples (HC, H45, and PC).

The electrical resistivity of single GNP, single CNT, and 50:50 GNP/CNT at 6 wt.% with different processing is plotted in Figure 7. The nanocomposite filled with only GNP was an insulating material, having a resistivity higher than 10^13^ Ω∙cm, independent of the processing. The resistivity of GNP/CNT (50:50) hybrid nanocomposites was 8.45 Ω∙cm, slightly higher than that of the nanocomposite with only CNT (ρ = 4.1 Ω∙cm), analogously to the correspondent compression molded samples. Moreover, much higher resistivity of GNP/CNT (50:50) hybrid nanocomposites was determined for FFF samples, at about 4.2 × 10^5^ Ω∙cm, 1.5 × 10^5^ Ω∙cm, and 1.1 × 10^4^ Ω∙cm for HC, H45, and PC, respectively. Following these results, nanocomposite FFF plates with 6 wt.% of CNT were successfully produced for strain monitoring applications [47].

### 3.4. Electromagnetic Interference Shielding Effectiveness (EMI SE)

**Figure 8:**
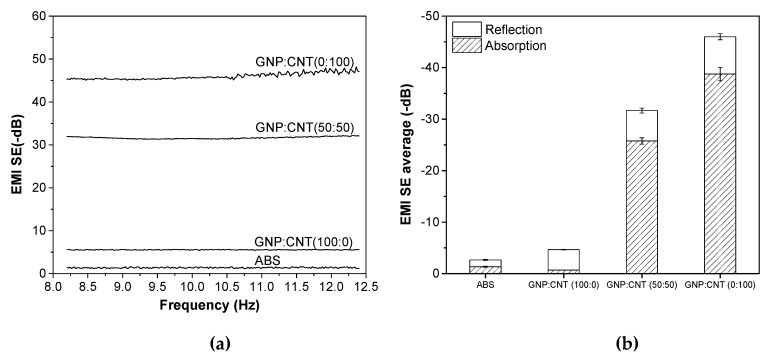
Electromagnetic interference shielding effectiveness (EMI SE0 of neat ABS, as well as single and hybrid nanocomposites, produced by compression molding with a total filler content of 6 wt.%: (**a**) representative curves and (**b**) influence of absorption SE_A_ and reflection SE_R_ mechanisms.

Electromagnetic interference shielding effectiveness (EMI SE) is determined as the shielding ability of materials from electromagnetic waves. Figure 8 shows the representative plots of EMI SE expressed in decibel (dB) in the frequency range from 8 to 12.4 GHz of the neat ABS and various single GNP, single CNT, and hybrid (50:50) nanocomposites at 6 wt.% produced by compression molding. The EMI SE of all these samples was almost independent of the frequency. The higher shielding effectiveness was achieved in the order of GNP/CNT (0:100) > (50:50) >> (100:0) > ABS samples. These results show a good correlation to electrical volume resistivity. Materials for EMI shielding purposes are generally targeted to have a minimum of −20 dB of attenuation, because, at these values of shielding, more than 99% of the incident wave is attenuated, ensuring that electronic equipment does not generate or is not affected by electromagnetic interference [48,49]. Therefore, it is interesting to note that samples containing 6 wt.% of CNT and hybrid (50:50) nanofillers could reach EMI SE levels of −46 dB and −31.7 dB, respectively, which meet the EMI SE levels required for commercial applications.

The average values of reflection and absorption contributing in the frequency range from 8.2–12.4 GHz are compared in Figure 8b. It is evident that the shielding absorption contribution SE_A_ of composites containing carbon nanotubes was higher than that of the reflection, i.e., SE_A_ > SE_R_. On the other hand, the dominant shielding mechanism for ABS/graphene composites was reflection, due to the platelet-shaped GNP that provided a higher surface area for interaction with the electromagnetic waves.

**Figure 9:**
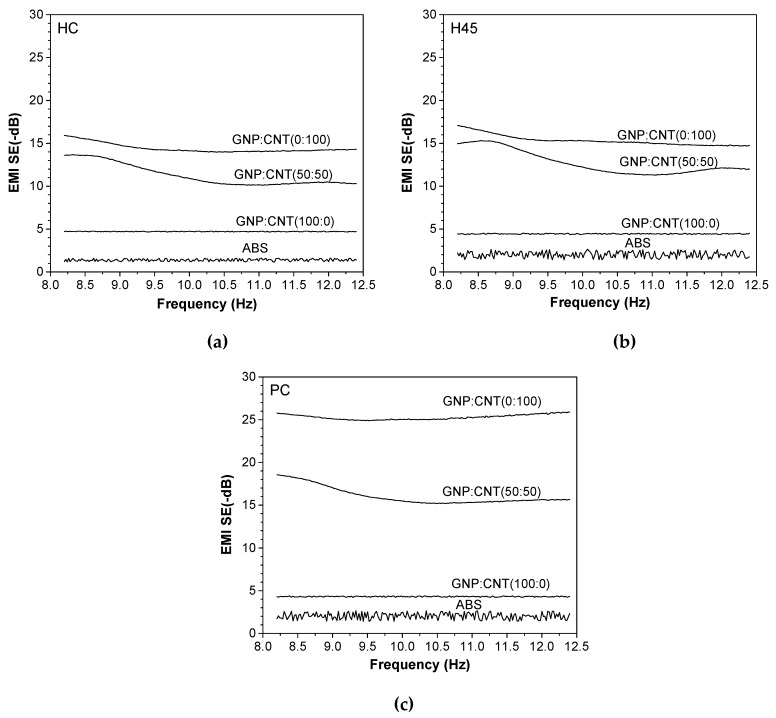
Representative curves of EMI SE of hybrid nanocomposite samples produced by FFF three-dimensional (3D) printing: (**a**) HC, (**b**) H45, and (**c**) PC.

Figure 9a–c show the representative plots of EMI SE of the neat ABS and various single GNP, single CNT, and hybrid (50:50) nanocomposites at 6 wt.% produced by FFF at different build orientations in the frequency range from 8 to 12.4 GHz. The nanocomposites with CNT show a slight influence of the shielding effectiveness on the frequency in the X-band. Accordingly, EMI SE responses were found to be a function of both the type of filler and the build orientation of the specimens. The shielding effectiveness decreased in the following order: GNP/CNT (0:100) > (50:50) >> (100:0) > ABS, independently of the build orientation of the specimens. These results show the same tendency as the electrical volume resistivity. Regarding the build orientation, it can be observed that the specimens prepared along the PC build orientation exhibited a better attenuation (of the electromagnetic radiation). For instance, the total EMI SE of the carbon nanotube-based composite built along PC was around −25.3 dB, whereas the same composite built along HC and H45 exhibited attenuation of −14.4 and −15.3 dB, respectively. Similar differences can also be observed for hybrid composites, with EMI SE values of −16.1, −11.3, and −12.7 dB for PC, HC, and H45, respectively. On the other hand, ABS/GNP composites, directly dependent on their high level of resistivity, revealed poor values of attenuation, near −4.5 dB, independent of the build direction.

**Figure 10:**
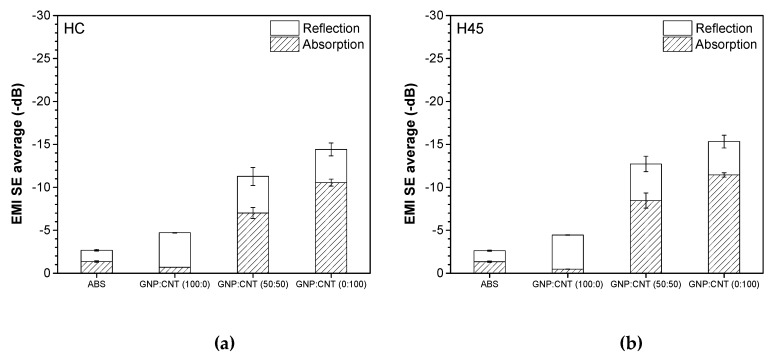

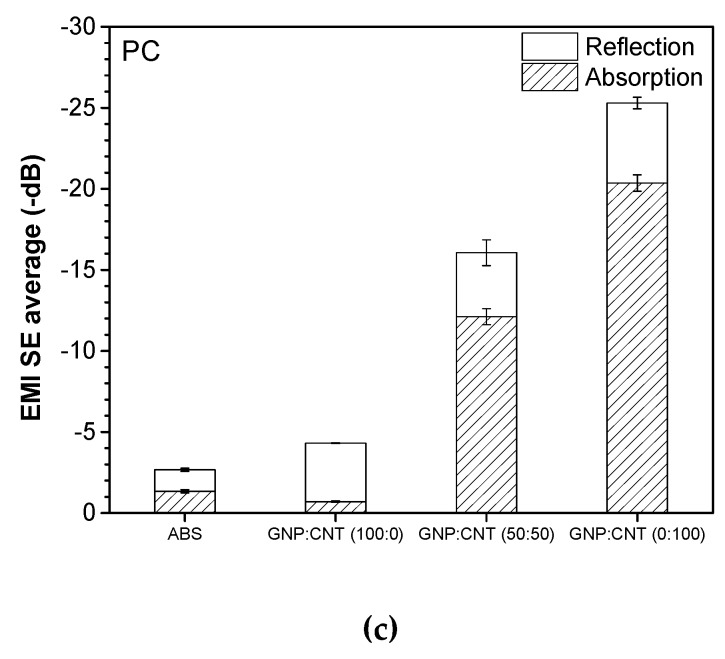
Influence of absorption and reflection mechanisms on the EMI SE of hybrid nanocomposites from FFF 3D printing: (**a**) HC, (**b**) H45, and (**c**) PC.

The average values of reflection and absorption contributions in the frequency range of 8.2–12.4 GHz are reported in Figure 10. The results of FFF samples regardless of the build orientation were analogous to those of compression molded samples. In fact, the shielding absorption contribution in nanocomposites containing carbon nanotubes was higher than that of the reflection, whereas the dominant shielding mechanism was reflection for ABS/graphene composites.

**Figure 11:**
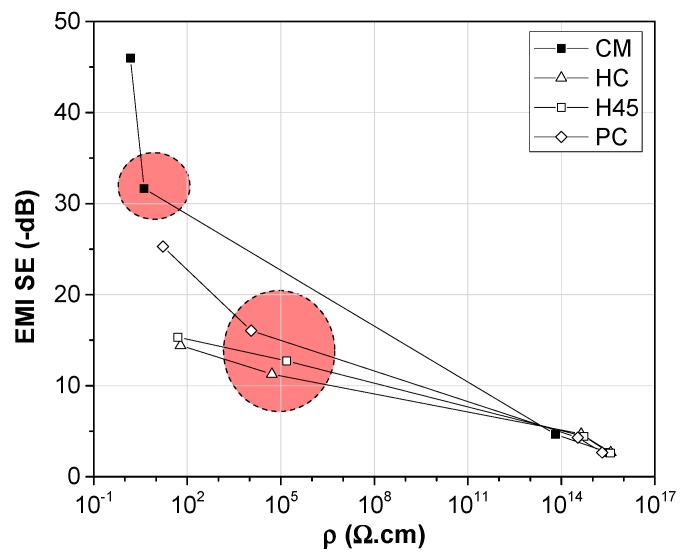
Electromagnetic shielding vs. resistivity of neat ABS, and different GNP, CNT, and hybrid nanocomposites (red circles) at 6 wt.% produced by compression molding (CM) or by 3D printing.

The relationship between the decrease in resistivity and the correspondent effect of increasing the magnetic shield after the addition of carbonaceous fillers is summarized in Figure 11. The better performance of CNT and GNP/CNT hybrid nanocomposites, with reduced resistivity and the correspondent higher EMI SE, is quite evident. The samples produced by compression molding evidence better results than those obtained from 3D printing.

### 3.5. Comparison of the Results to Literature Data

**Figure 12:**
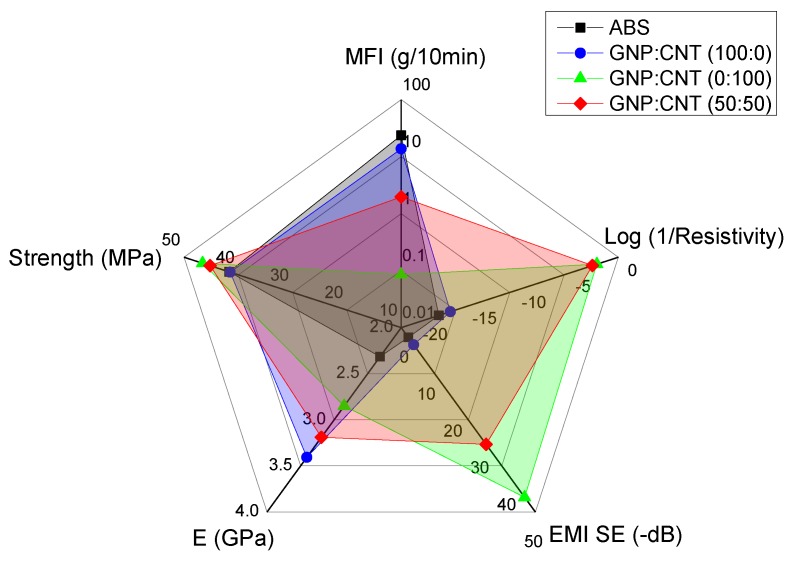
Comparative plot of compression molded nanocomposite at 6 wt.%. Processability (MFI), resistivity, electromagnetic shielding (EMI SE), and tensile properties of graphene, carbon nanotube, and 50:50 hybrid nanocomposites with respect to ABS matrix. Spider plots of FFF samples are reported in Figure S3 (Supplementary Materials) for comparison.

This paragraph summarizes the main results of these studied GNP/CNT nanocomposites, and compares their values with some relevant literature data. Figure 12 shows the processability (melt flow index), resistivity, electromagnetic shielding, and tensile properties of graphene, carbon nanotube, and hybrid nanocomposites at 6 wt.% from compression molding. It is interesting to observe that the GNP/CNT (50:50) samples were a good compromise between the decrease in processability and the increase in mechanical, electrical resistivity, and electromagnetic properties. Analogous pictures were found for FFF samples produced with different build orientation, as shown in Figure S2 (Supplementary Materials). In particular, PC samples exhibited better conductive and EMI SE properties with respect to HC and H45 samples, but lower mechanical properties. 

The summary of the main properties of GNP/CNT hybrid nanocomposites at 6 wt.% is also reported in Table S1 (Supplementary Materials). For the purpose of quantitative evaluation of the effect of GNP/CNT relative ratio, we can determine comparative parameters that take into consideration some specific properties important for applications. In particular, the properties of nanocomposites such as the stiffness, the processability, and the electrical resistivity should be taken into account. Hence, an interesting merit parameter *P_E,M,ρ_*was recently defined [40]. From Figure S2 (Supplementary Materials), the *P_E,M,ρ_* parameter assumes the highest value at 50:50 and 70:30 GNP/CNT relative ratios. 

A final comparison of properties of the thermoplastic nanocomposites presented in this study and some correspondent engineering polymers containing similar types of carbon nanoparticles is detailed in Table 3. The processing techniques and the comparative values could be useful for ranking the various nanocomposites in terms of dependence on the required applications. The addition of carbonaceous nanofillers is reported to provide beneficial effects in reinforcement and in electrical performances of the various thermoplastics, such as ABS, ABS/polycarbonate (PC), PLA, polybutylene terephthalate (PBT), polyamide 12 (PA12), PHA (polyhydroxy alkanoate), and PEEK. CNT provides a significant increase in electrical and electromagnetic shielding properties, much higher than GNP with a similar amount of filler. For instance, on ABS/CNT composites with 5 wt.% of filler content, electromagnetic shielding of −38.0 dB at 8.0–12.0 GHz and electrical conductivity values of and 2.0 × 10^−3^ S/cm were reached [50]. An EMI SE value of −60 dB at 8.0–12.0 GHz and conductivity of 0.166 S/cm for ABS composites were achieved by adding 15 wt.% of graphene nanoplatelets [51]. The properties of the products from FFF show sometimes higher properties than those produced by compression molding. Spinelli et al. [33] produced PLA nanocomposites containing 12 wt.% of GNP, CNT, and their mixing for 3D printing. The 3D-printed parts exhibited improved electrical conductivity up to 4.54 S/m, 6.57 S/m, and 0.95 S/m respectively. EMI SE values in the frequency range 26–37 GHz of samples also increased from 0.20 dB of unfilled PLA up to −13.4 dB for GNP/CNT (1:1) nanocomposites. However, taking into consideration the T_g_ of the PLA matrix, a specific application temperature is in the range 20–50 °C, which is relatively lower than that of ABS (up to 80–90 °C). Another study reported ABS-based nanocomposites with the presence of hybrid carbon nanotube/carbon black (1:1) of 3 wt.% fillers from 3D printing with an EMI SE value of −8 dB in the range 8.2–12.4 GHz [22]. For high-performance thermoplastics, 3D-printed PEEK filled with 7 wt.% of a GNP/CNT (4:3) mixture demonstrated electrical conductivity of about 1.0 × 10^-6^ S/cm, but no electromagnetic shielding behavior was reported [35]. In our work, the ABS matrix was incorporated at 6 wt.% of GNP, CNT, and GNP/CNT (50:50) composites for 3D printing with EMI SE values in the frequency band 8.2–12.4 GHz of −4.4, −15.3, and −12.7 dB, respectively. 

## 4. Conclusions

The objective of the present work was to develop electrically conductive carbon-based thermoplastic materials, with good mechanical properties, suitable for 3D printing by fused deposition modeling. The manufacture of the nanocomposites with a total amont of 6 wt.% of GNP/CNT nanofiller was performed by direct melt compounding, and various samples with different compositions were produced either by compression molding or by filament extrusion. A higher CNT content led to lower resistivity and higher electromagnetic shielding, but lower melt flow index. The GNP/CNT hybrid nanocomposites showed values of elastic modulus, strength, electrical resistivity, and processability intermediate to those manifested by nanocomposites filled with either GNP or CNT. Substitution of GNP by CNT provided a positive effect on the electrical resistivity and an improvement of EMI SE, but a certain reduction of modulus and flow properties. The electrical conductive filament (ρ = 8.5 Ω∙cm) and FFF parts were achieved after the addition of GNP/CNT content. However, their resistivity increased after the 3D printing process. GNP/CNT hybrid composition of 6 wt.% carbonaceous nanocomposites showed a good compromise between processability and enhancement of properties (mainly mechanical and electrical properties). In agreement with electrical resistivity, EMI SE of 6 wt.% ABS/CNT and 50:50 hybrid ABS nanocomposites resulted as −46 dB and −31.7 dB for plate samples. The EMI SE of FFF parts was about −14 dB for HC and H45 build orientations and −25 dB for the PC build orientation for ABS/CNT nanocomposites. Similar EMI SE values of FFF hybrid nanocomposites were observed, almost independent of the building process, of about −12 dB for HC and H45, and −16 dB for PC samples.

## Figures and Tables

**Table 1:** Properties of commercial grades of graphene nanoplatelets (GNPs) and multi-walled carbon nanotubes (CNTs) according to the manufacturer.

Nanoparticle	Length/Width (μm)	Diameter/Thickness (nm)	Surface Area (m^2^/g)	Carbon Purity (%)	Density (g/cm^3^)	Manufacturer
xGnP-M5	5	6–8	120–150	>99.5	2.06 ± 0.03 *	XG sciences, USA [42]
MWCNT-NC7000	1.5	9.5	250–300	>90	2.15 ± 0.03 **	Nanocyl, Belgium [43]

* Data extracted from Reference [44]; ** data extracted from Reference [39].

**Table 2:** Designation and formulation of acrylonitrile butadiene styrene (ABS) hybrid nanocomposites at different GNP/CNT ratios.

Sample	ABS (wt.%)	GNP (wt.%)	CNT (wt.%)	GNP/CNT Relative Ratio
ABS	100	0	0	0:0
GNP/CNT (100:0)	94	6.0	0.0	100:0
GNP/CNT (90:10)	94	5.4	0.6	90:10
GNP/CNT (70:30)	94	4.2	1.8	70:30
GNP/CNT (50:50)	94	3.0	3.0	50:50
GNP/CNT (30:70)	94	1.8	4.2	30:70
GNP/CNT (10:90)	94	0.6	5.4	10:90
GNP/CNT (0:100)	94	0.0	6.0	0:100

**Table 3:** Comparison of selected properties of ABS nanocomposites studied in this research with respect to other carbon-based engineering polymers.

Matrix	Type of Nanofiller	Nanofiller	Process Technique *	Modulus **	Strength **	Conductivity (S/cm)	EMI SE		References
		Content					Thickness	(-dB)	
	Graphene								
ABS	GNP nanosheet	0.13 vol.%	SM/CM	-	-	1.0 × 10^−3^	-	-	[52]
ABS	C18-graphene	1 wt.%	SM/SC	+18%	+38%	−	-	-	[53]
ABS	GNP nanosheet	2 wt.%	SM/CM	+48%	+41%	−	-	-	[54]
ABS	Graphite	4.9 vol.%	MM/CM	-	-	2.0 × 10^−1^	-		[55]
ABS	GO	5.6 wt.%	SM/3DP	-	-	1.1 × 10^−5^	-	-	[56]
ABS	Graphite	15 wt.%	MM/CM	-	-	1.6 × 10^−1^	3 mm	60 at 8.0–12.0 GHz	[51]
ABS	Graphite	40 vol.%	MM/IM	+96%	−19%	−	-	-	[57]
ABS/PC	GNP	3 wt.%	MM/IM	+30%	+15%	−	-	-	[58]
PLA	r-GO	6 wt.%	MM/3DP	+36%	+74%	4.7 × 10^0^		-	[59]
PLA	GNP	6 wt.%	MM/3DP	-	-	8.4 × 10^−5^	-	-	[31]
PLA	GNP	12 wt.%	MM/3DP	-	-	6.3 × 10^−2^	10 mm	10.2 at 30 GHz	[33]
PBT	GNP	8.4 vol.%	SM/3DP	-	-	4.0 × 10^−2^	-	-	[60]
PA12	Graphene	5 wt.%	MM/CM	-	-	2.0 × 10^−2^	-	-	[61]
	Carbon Nanotubes								
ABS	CNT	3 wt.%	MM/CM	-	-	10^−2^	2 mm	10 at 8.2–12.4 GHz	[22]
ABS	CNT	5 wt.%	Solid mixing/CM	-	-	2.0 × 10^−3^	2.8 mm	38.0 at 8–12 GHz	[50]
ABS	CNT	6.1 vol.%	SM/CM	-	-	1.0 × 10^0^	-	-	[62]
PLA	CNT	12 wt.%	MM/3DP	-	-	4.5 × 10^−2^	10 mm	10.2 at 30 GHz	[33]
PLA	CNT	6 wt.%	MM/3DP	-	-	2.1 × 10^−4^	-	-	[31]
PHAs	f-MWCNT	1 wt.%	MM/extrusion	+33%	+102%	1.0 × 10^−7^	-	-	[63]
PA12	CNT	5 wt.%	MM/CM	-	-	1.4 × 10^−1^	-	-	[61]
PBT	CNT	3.5 vol.%	SM/3DP	-	-	2.5 × 10^−1^	-	-	[60]
	Carbon Black								
ABS	CB	3 wt.%	MM/CM	-	-	10^−7^	2 mm	4 at 8.2–12.4 GHz	[22]
ABS	CB	15 wt.%	MM/3DP	-	-	3.4 × 10^−4^	-	-	[64]
	Hybrids								
ABS	CB/CNT	3 wt.%	MM/3DP	-	-	10^−3^	2 mm	8 at 8.2–12.4 GHz	[22]
PLA	GNP/CNT	12 wt.%	MM/3DP	~20%	-	−	-	-	[46]
PLA	GNP/CNT	12 wt.%	MM/3DP	46%	-21%	2.2 × 10^−3^	-	-	[65]
PLA	GNP/CNT	6 wt.%	MM/3DP	-	-	5.9 × 10^−2^	-	-	[31]
PLA	GNP/CNT	12 wt.%	MM/3DP	-	-	9.5 × 10^−3^	10 mm	13.5 at 30 GHz	[33]
PEEK	GNP/CNT	7 wt.%	MM/3DP	~ +11%	~ +2%	~1.0 × 10^−6^	-	-	[35]
ABS	-	0	MM/3DP	2308 MPa	41.1 MPa	1.6 × 10^−16^	2 mm	2.7 at 8.2–12.4 GHz	This study
ABS	GNP	6 wt.%	MM/3DP	+38%	−1%	1.9 × 10^−15^	2 mm	4.4 at 8.2–12.4 GHz	This study
ABS	CNT	6 wt.%	MM/3DP	+19%	+5%	6.8 × 10^−6^	2 mm	15.3 at 8.2–12.4 GHz	This study
ABS	GNP/CNT	6 wt.%	MM/3DP	+38%	−4%	2.0 × 10^−2^	2 mm	12.7 at 8.2–12.4 GHz	This study

* MM is melt mixing; SM is solution mixing; CM is compression molding; IM is injection molding; SC is solution casting; 3DP is three-dimensional printing. ** Relative percentage variation with respect to the neat polymeric matrix. GO: graphene oxide; r-GO: reduced graphene oxide; f-MWCNT: functionalized multi-walled carbon nanotubes; PC: polycarbonate; PLA: polylactide;PBT: polybutylene terephthalate; PA12: polyamide 12; PHAs: polyhydroxy alkanoate); PEEK: polyether ether ketone.

## Supplementary Materials

The following are available online at https://www.mdpi.com/2073-4360/12/1/101/s1: Figure S1. Representative stress–strain curves of neat ABS, and GNP/CNT (100:0), GNP/CNT (50:50), and GNP/CNT (0:100) nanocomposites: (a) compression molded samples, (b) filaments, (c) HC, (d) H45, and (e) PC samples; Figure S2. The merit parameter P from Equation (S1) combines and compares the effects of elastic modulus, melt flow index (220 °C), and resistivity of nanocomposites with CNT/GNP 6 wt.% as a function of relative ratio of CNT (from Table S1); Figure S3. Spider plots of FFF samples with relative comparison of processability (MFI), resistivity, electromagnetic shielding (EMI SE), and tensile properties of graphene, carbon nanotube, and 50:50 hybrid nanocomposites with respect to the ABS matrix: (a) HC, (b) H45, and (c) PC; Table S1: The summary of properties of GNP/CNT hybrid nanocomposites.
