# Supplementary material for: Graphene/Carbon Nanotube Hybrid Nanocomposites: Effect of Compression Molding and Fused Filament Fabrication on Properties
Source: Polymers (Basel). 2020 Jan 4;12(1):101. doi: 10.3390/polym12010101 (PMC7022553; doi:10.3390/polym12010101)
Supplement: Supplementary file 1 [file polymers-12-00101-s001.pdf]

Supplementary Materials: Graphene/Carbon Nanotubes Hybrid Nanocomposites: Effect of Compression Molding and Fused Filament Fabrication on Properties

Sithiprumnea Dul, Luiz Gustavo Ecco, Alessandro Pegoretti, Luca Fambri *

List of Supplementary Materials:

Figure S1. Representative stress-strain curves of neat ABS, and GNP:CNT (100:0), GNP:CNT (50:50), GNP:CNT (0:100) nanocomposites: (a) compression moulded samples, (b) filaments, (c) HC, (d) H45 and (e) PC samples.

Figure S2. The merit parameter PE,M, from Eq. S1 combines and compares the effect of elastic modulus, melt flow index (220°C) and resistivity of nanocomposite with CNT/GNP 6 wt% as a function of relative content of CNT (from Table S1).

Figure S3. Spider plots of FFF samples with relative comparison of processability (MFI), resistivity, electromagnetic shielding EMISE), and tensile properties of graphene, carbon nanotubes and 50:50 hybrid nanocomposites with respect to ABS matrix: (a) HC, (b) H45 and (c) PC.

Table S1. Comparison of selected properties of GNP:CNT hybrid nanocomposites with 6% wt (melt compounded and compression molded samples).

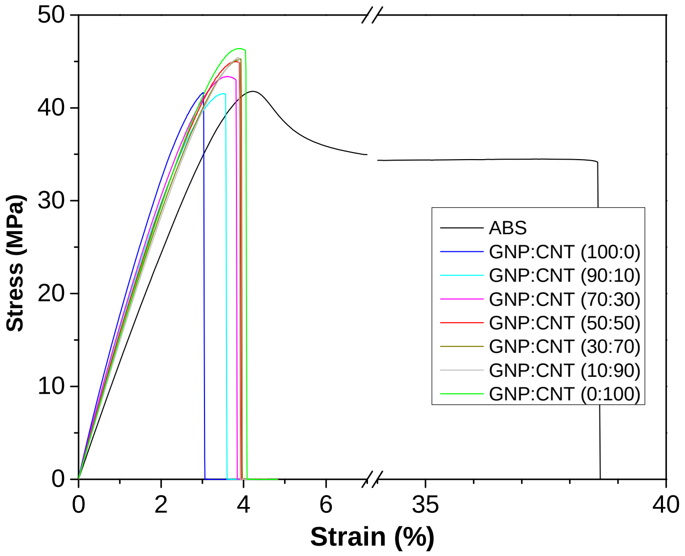

(a)

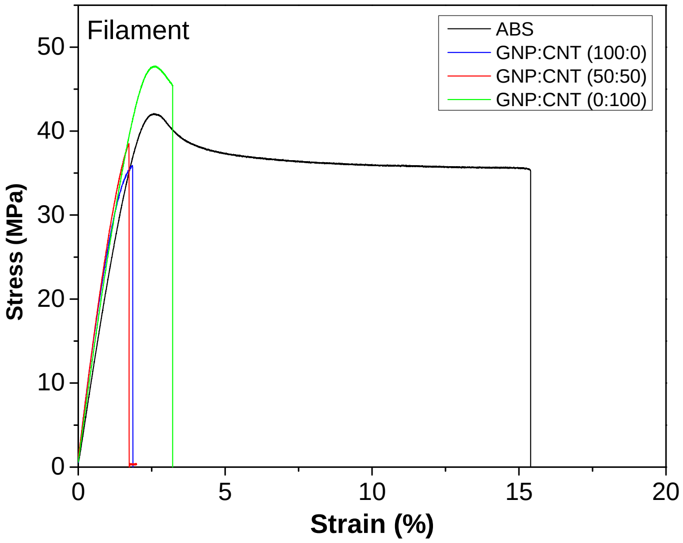
Filament
ABS
GNP:CNT (100:0)
GNP:CNT (50:50)
GNP:CNT (0:100)
Stress (MPa)
Strain (%)
0
10
20
30
40
50
0
5
10
15
20

(b)

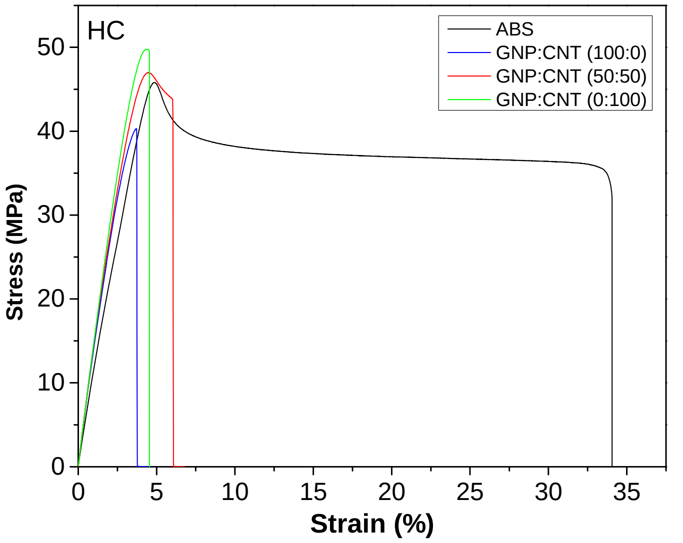
HC
ABS
GNP:CNT (100:0)
GNP:CNT (50:50)
GNP:CNT (0:100)
Stress (MPa)
Strain (%)
0
10
20
30
40
50
0
5
10
15
20
25
30
35

(c)

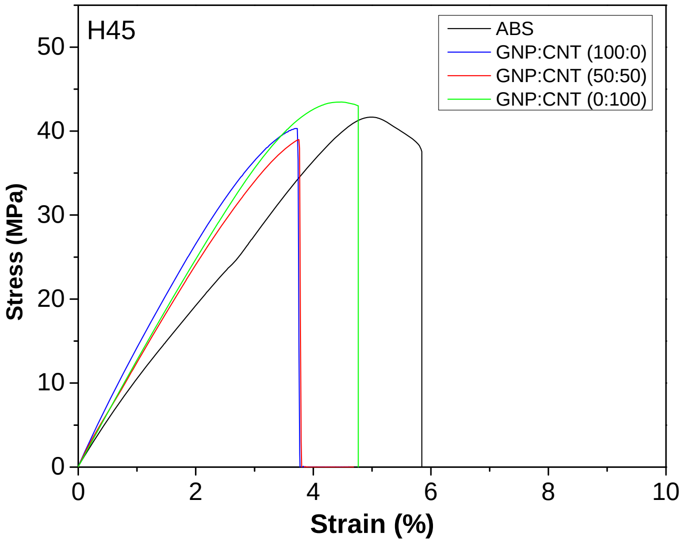

(d)

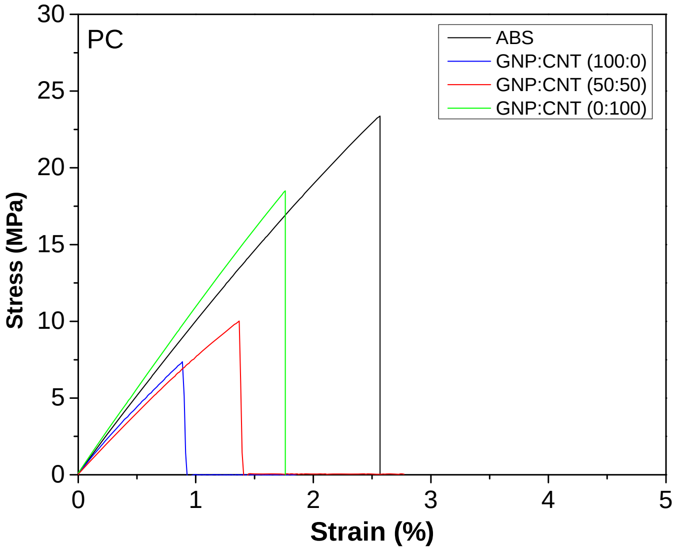

(e)

Figure S1. Representative stress-strain curves of neat ABS, and GNP:CNT (100:0), GNP:CNT (50:50), GNP:CNT (0:100) nanocomposites: (a) compression moulded samples, (b) filaments, (c) HC, (d) H45 and (e) PC samples.

The effect of GNP/CNT relative ratio can be quantitative evaluated by the comparative merit parameter $P_{E,M,\rho}$ taking into account the stiffness, the processability and the conductivity of ABS and its GNP:CNT composites. In particular the merit parameter $P_{E,M,\rho}$ has been previously defined [1] according to Equation (S1):

$$P_{E,M,\rho} = \text{E x MFI}/\rho \qquad \text{(Eq. S1)}$$

where E is the modulus, MFI is the melt flow index at 220°C/10kg and ρ is the resistivity. All data are reported in **Table S1**, and the resulting values are depicted in **Figure S2**.

Table S1. Comparison of selected properties of GNP:CNT hybrid nanocomposites with 6% wt (melt compounded and compression molded samples).

**Relative ratio** GNP-CNT	**MFI (g/10min)**	**ρ (Ω.cm)**	**E (MPa)**	**Strength (MPa)**	**ε_b (%)**	**TEB ($MJ.mm^{-3}$)**	**$P_{E,M,\rho}$ = E × MFI/ρ**
0:0 *	23.61	3.27E+15	2315	41.7	35.9	11.785	1.67E-11
100:0	13.71	1.04E+15	3406	41.5	3.1	0.788	7.25E-10
90:10	9.77	1.49E+07	3338	41.7	3.5	0.868	2.19E-03
70:30	5.22	1.27E+01	3275	43.2	3.8	0.995	1.35E+03
50:50	1.97	4.13E+00	3189	45.2	4.1	1.156	1.52E+03
30:70	0.42	1.90E+00	3064	45.2	4.1	1.110	6.75E+02
10:90	0.09	1.54E+00	2899	45.7	3.9	1.030	1.68E+02
0:100	0.08	1.51E+00	2849	46.6	3.9	1.112	1.60E+02

*neat ABS

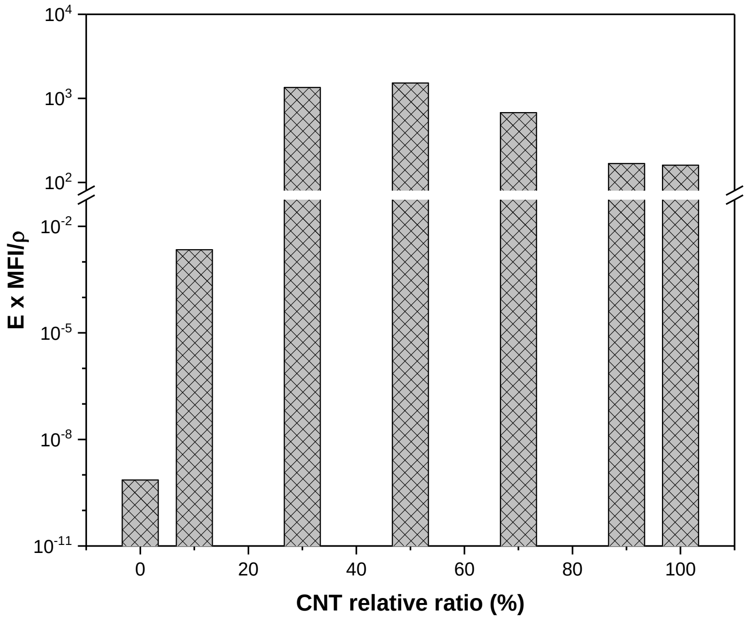

Figure S2. The merit parameter $P_{E,M,\rho}$ from **Eq. S1** combines and compares the effect of elastic modulus, melt flow index (220°C) and resistivity of nanocomposite with CNT/GNP 6 wt% as a function of relative content of CNT (from **Table S1**).

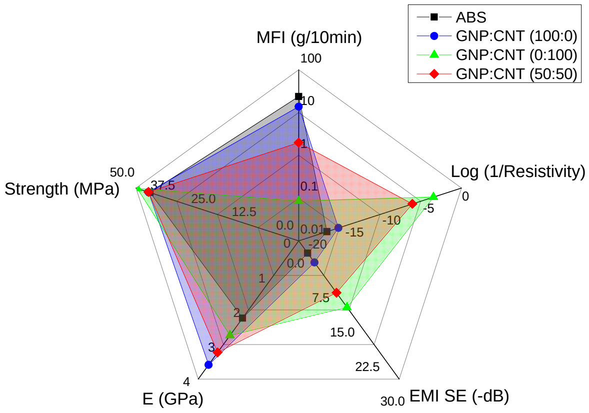

(a) HC

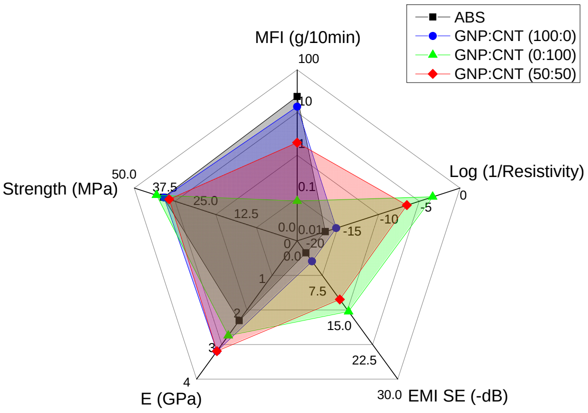

(b) H45

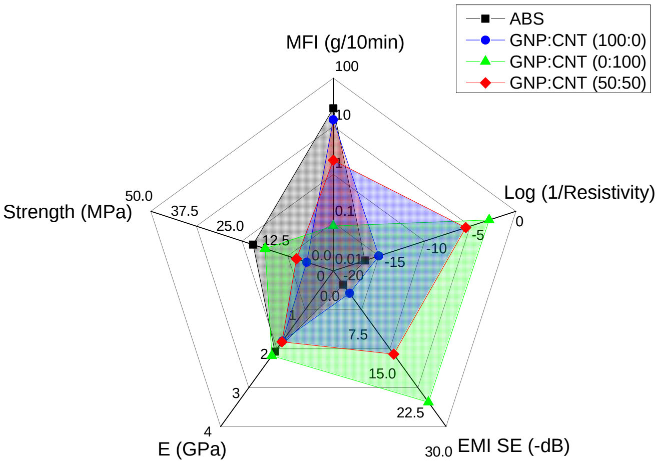

(c) PC

Figure S3. Spider plots of FFF samples with relative comparison of processability (MFI), resistivity, electromagnetic shielding EMISE), and tensile properties of graphene, carbon nanotubes and 50:50 hybrid nanocomposites with respect to ABS matrix: (**a**) HC, (**b**) H45 and (**c**) PC.

Reference

1. Dul, S.; Pegoretti, A.; Fambri, L. Effects of the nanofillers on physical properties of acrylonitrile-butadiene-styrene nanocomposites: Comparison of graphene nanoplatelets and multiwall carbon nanotubes. *Nanomaterials* **2018**, *8*, 674-693.
